# Early neuro-electric indication of lexical match in English spoken-word recognition

**DOI:** 10.1371/journal.pone.0285286

**Published:** 2023-05-18

**Authors:** Pelle Söderström, Anne Cutler

**Affiliations:** 1 Centre for Languages and Literature, Lund University, Lund, Sweden; 2 MARCS Institute for Brain, Behaviour & Development, Western Sydney University, Penrith, Australia; 3 ARC Centre of Excellence for the Dynamics of Language, St Lucia, Australia; 4 Max Planck Institute for Psycholinguistics, Nijmegen, Netherlands; Max-Planck-Institut fur Kognitions- und Neurowissenschaften, GERMANY

## Abstract

We investigated early electrophysiological responses to spoken English words embedded in neutral sentence frames, using a lexical decision paradigm. As words unfold in time, similar-sounding lexical items compete for recognition within 200 milliseconds after word onset. A small number of studies have previously investigated event-related potentials in this time window in English and French, with results differing in direction of effects as well as component scalp distribution. Investigations of spoken-word recognition in Swedish have reported an early left-frontally distributed event-related potential that increases in amplitude as a function of the probability of a successful lexical match as the word unfolds. Results from the present study indicate that the same process may occur in English: we propose that increased certainty of a ‘word’ response in a lexical decision task is reflected in the amplitude of an early left-anterior brain potential beginning around 150 milliseconds after word onset. This in turn is proposed to be connected to the probabilistically driven activation of possible upcoming word forms.

## 1. Introduction

Listening to spoken language presents listeners with the formidable task of re-interpreting a continuous stream of speech as a sequence of separate words; this conversion is the only way that we can begin to understand our interlocutor’s message. Words unfolding in time compete with similar-sounding words, and in this process of lexical competition, listeners rapidly entertain multiple hypotheses about the possible identity of each incoming word. For example, the English word *service* shares its first two speech sounds ([sɜː]) with five times as many possible words as compared to *nervous* ([nɜː]): thus [sɜː] can go on to form *Serbian*, *service*, *certainty*, *surcharge*, *sirloin* and more, while [nɜː] cues a much smaller set of possible words (*nervous*, *nurture*, *nursing* [1]).

The competition process becomes active within 200 milliseconds from the onset of each word in the incoming stream [2], and has been suggested to proceed according to probabilistic principles [3]. The baseline prior probability that a particular word will be heard is reflected in the frequency at which it occurs in the language, but this prior probability can also be changed by local or global contexts. For example, *car* is a more frequent word than *par* [1], but the probability of its occurrence is likely to change in a conversation about golf.

At a lower, pre-lexical level, the probability of encountering particular phonemes within a word is controlled by other prior probabilities, namely those provided by preceding speech sounds in the signal [3,4]: since far fewer words begin with [zɛ] as in *zealous* as compared to [ʤɛ] in *jealous*, the probability of a lexical match may increase more sharply in the former case.

In these ways, the first few speech sounds in an incoming utterance constitute a micro-context which allows a listener to narrow the decision space as to the identity of the unfolding word. There is as yet no agreed picture of the early neuro-electric correlates of this process, however. This most likely arises from the existing literature, in that the electroencephalographic studies of spoken-word recognition carried out so far–some in French and some in English, as reviewed below–have differed in both their experimental design and the interpretations provided of the observed effects.

In the present study, we use electroencephalography (EEG) to investigate event-related potentials (ERPs) associated with spoken-word recognition and lexical competition in this early time window (150–200 ms from word onset). Our analyses draw on a body of work investigating spoken-word recognition in Swedish and Danish [5–14], where several studies have found an early ERP component which has been suggested to reflect the graded probability of successfully predicting the ending of a word. We propose that this component may be a language-non-specific neuro-electric reflection of the process whereby the evidence for a certain lexical hypothesis or decision increases.

The previous studies on spoken French and English words have mainly focused on the question of whether early neuro-electric responses reflect facilitatory or inhibitory effects of lexical competition at the sub-lexical or lexical level, but results and interpretations have differed.

A study of spoken French monosyllabic words suggested that phonological neighbourhood density–the number of words differing from a given word by a single phoneme [15–17]–facilitates word recognition. Words in more dense neighbourhoods elicited a smaller ERP negativity in a 250–330 ms time window after word onset, with a broad, bilateral frontal distribution [18]. This ERP effect was viewed as a modulation of the phonological mismatch (or mapping) negativity (PMN), an effect originally found in response to phonological violations, i.e., unexpected word-initial phoneme substitutions in otherwise expected words [19–21]. Based on this assumption, the reduced negativity was interpreted as a facilitatory effect of denser neighbourhoods at the pre-lexical phonemic level of processing.

Hunter [22] found ERP effects of neighbourhood density in English monosyllabic words going in the opposite direction, and with a differing topographic profile: in two different tasks, dense neighbourhoods were associated with *positivities* between 200–300 ms that displayed a posterior scalp distribution. These were interpreted as amplitude increases in the P2 component. This interpretation was based on findings from visual-word recognition research [23–25], where it had been suggested that P2 amplitude differences are a reflection of high-density stimuli taxing neural resources more heavily, due to lateral inhibition or increased lexical activation between candidates. However, due to the differences between spoken and visual word recognition, it may be difficult to draw parallels between them in the interpretation of ERP components. In a subsequent study, Hunter [26] used phonological neighbourhood as dependent variable in a lexical decision task, while *controlling* for cohort size (i.e., the number of possible words beginning with a particular onset). No ERP effects of phonological neighbourhood density earlier than the N400 appeared. This null effect could suggest, however, that cohort size indeed might modulate early ERP amplitudes.

A study of English mono- and disyllabic words [27] found more negative ERP amplitudes for dense neighbourhoods over bilateral central electrodes, 200–300 ms after word onset. The effect was interpreted as a reflection of large cohorts of words co-activating sub-lexical or lexical networks, facilitating early-stage word recognition. It was suggested that phonotactic probability and neighbourhood density may have opposite effects on ERP amplitude, and that the early positivities found in previous studies could instead be explained as a facilitatory effect of increased phonotactic probability and connections between sub-lexical units rather than an inhibitory effect of increased lexical competition.

Previous studies in French and English thus show differing findings and interpretations of early ERP effects in spoken-word recognition, with the situation made more complicated by the comparison of results between visual and spoken-word recognition, as well as reference to mismatch components in non-mismatch paradigms. However, early ERP responses to spoken word onsets have also been investigated in Scandinavian languages, with a more consistent pattern of results that may shed a light on the early underlying processes in spoken-word recognition. A large number of studies have found that word onsets with fewer lexical competitors elicit an early, left-frontal ERP negativity, which has in essence been interpreted as reflecting a facilitatory role of reduced lexical competition. In Swedish, every word has a lexical stem tone, which is largely determined by the morphological structure of the word. Adding a singular suffix (*-en*) to the word stem *båt* (‘boat’) renders one tone (‘accent 1’) on the stem (*båt*_*1*_*-en*, ‘the boat’), while a plural suffix (*-ar)* assigns another tone (‘accent 2’) to the word stem: *båt*_*2*_*-ar* (‘boats’). Monosyllabic words have accent 1 by default. Stem tones can thus carry clues as to how the word is going to end. Using tasks where participants judge whether the word is e.g. singular/plural as quickly as possible, it has been shown that listeners take advantage of these regularities to predict upcoming word endings, using the stem tone as a clue, and word onsets with fewer possible continuations elicit larger ERP negativities [5–11,13,28]. Importantly for the present discussion, all Swedish compound words–a highly productive lexical category–are assigned accent 2 on the first syllable. Thus, word onsets with accent 2 have on average 11 times as many possible word continuations compared to accent 1 stems [14] and consequently lead to a large increase in lexical competitors. Word onsets with accent 1 have fewer possible continuations, meaning that it is easier for the listener to predict the upcoming word ending. This increased certainty has been found to be reflected in the amplitude of a brain potential referred to as the pre-activation negativity (PrAN) [5–10,13,28,29]. With a left-lateralised frontal topography, PrAN normally begins around 150–200 ms after word onset. It correlates with activity in the primary and secondary auditory cortices–areas that play an important role in lexical predictions in accordance with the predictive coding framework [4]–as well as angular gyrus (Brodmann area 39) and left inferior frontal gyrus (Brodmann areas 44 and 47) [9,10]. The effect has been suggested to reflect the predictive strength of phonological cues: more predictively useful or informative cues give rise to increased PrAN amplitudes, facilitating subsequent processing [14,30]. Its amplitude decreases linearly with the number of possible continuations of the unfolding word and increases along with word frequency [7,14]. It has also been found to correlate negatively with increasing (segmental) phonological neighbourhood density as traditionally calculated (i.e., one-phoneme substitution [17,31]). Thus, denser phonological neighbourhoods elicit smaller early ERP negativities in Swedish. Also, since Swedish words can be segmentally identical and differ in stem tone only–cf. *anden*_*1*_ (‘the duck’) and *anden*_*2*_ (‘the spirit)–the effect of phonotactic probability on ERPs is effectively controlled. In sum, this early negativity has been interpreted as reflecting increased certainty of rapid word identification, with the certainty being driven by decreasing lexical competition on the word stem in the Swedish paradigms, which in turn allows the word ending to be predicted more strongly. It is unlikely, however, that PrAN reflects a uniquely Swedish process, and it may be useful to consider early negativities as broader indices of lexical match in spoken-word recognition. Lexical competition may influence ERP amplitudes–and indeed behavioural responses–differently depending on the experimental task. If the task is to essentially predict a word ending as quickly as possible, a word onset with fewer lexical candidates may rapidly increase certainty as to the ending, and consequently facilitate the process, leading to an increased early negativity. In a lexical decision task, however, increased lexical competition in the first phonemes may increase the listener’s confidence that a ‘word’ response will be successful, similarly to the interpretation of the early negativity in [27]. In the present study, we investigated early ERP effects elicited by English word onsets differing in the number of lexical competitors to shed further light on this issue. We hypothesised that lexical competition would modulate ERP amplitudes over left-anterior electrodes beginning around 150 ms after word onset [7], expecting to see a pre-activation negativity elicited under conditions that facilitate the early stages of spoken-word recognition. In Scandinavian languages, this has consistently been word onsets with fewer lexical competitors, whereas results are mixed in English and French-language studies. If–as suggested previously [27]–high-competition word onsets facilitate early word recognition and lexical match through the activation of lexical or sub-lexical networks in lexical decision tasks, one would expect that word onsets with high lexical competition elicit larger pre-activation negativities than onsets with low lexical competition.

## 2. Materials and methods

### 2.1 Stimulus materials

The target stimuli (two-syllable monomorphemic trochees) were recorded by a female native speaker of Australian English, who was instructed to pronounce the words as clearly as possible (see S1 File for stimulus list). The stimuli were recorded at a sample rate of 44.1 kHz, at 16 bits per sample. The words were embedded in carrier sentences (“She/he used the word [target] today.”), similarly to previous studies of Swedish [5–14,32]. Carrier sentence onset was counter-balanced across conditions. Low- and high-competition word pairs differed only in onset consonant (e.g. *gobble*/*cobble*) so as to control the effects of co-articulation as well as reduce the effect of phonotactic probability. Pseudoword pairs were created by replacing one (e.g. *gobble* > *gottle*) or two phonemes (*number* > *nunger*) in the original word pair. This method of creating pseudowords–along with constraints on being pronounceable and phonotactically legal in Australian English–may have led to an imbalance in how long they could have gone on to become words, something which may have an effect in the early stages of word recognition. Consequently, real word and pseudoword onsets were analysed separately. In total, there were 240 stimulus words in four conditions with 60 words each. These conditions are hereafter referred to as RealLo (real word, low competition), RealHi (real word, high competition), PseudoLo (pseudoword, low competition) and PseudoHi (pseudoword, high competition).

#### 2.1.1 Acoustic features

The word-initial fragments (calculated from word onset until onset of the second syllable) had an average duration of 415 ms (*SD* = 110), with no significant differences between real words and pseudowords (*t* = 0.237, *df* = 232.74, *p* = 0.813) or words with low and high competition (*t* = -0.111, *df* = 106.88, *p* = 0.912), as revealed by unpaired-samples *t*-tests. There were no significant differences between conditions in average intensity (dB SPL), or in midpoint measurements of first-syllable F0, F1 and F2 frequency. Mean total target word duration was 845 ms (*SD* = 130 ms), again with no significant differences between conditions (see Table 1 for details).

**Table 1:** Mean lexical and acoustic characteristics of target words.

	Low competition words	High competition words
Word onset competitors	**61.15** (4.03)	**290.90** (17.40)
Phonological neighbours	**8.43** (0.82)	**11.50** (1.16)
Log SUBTLEX-US frequency	1.95 (0.11)	2.12 (0.12)
Log SUBTLEX-UK frequency	3.32 (0.11)	3.41 (0.12)
Imageability (1–7)	5.17 (0.26)	4.64 (0.21)
Concreteness (1–5)	3.53 (0.14)	3.50 (0.15)
Age of acquisition (years)	8.87 (0.39)	8.98 (0.36)
1^st^ syllable duration (ms)	420.5 (2.1)	408.9 (2.5)
1^st^ syllable intensity (dB SPL)	67.8 (0.21)	69.3 (0.09)
1^st^ syllable F0 midpoint (Hz)	210.3 (0.62)	217.7 (0.63)
1^st^ syllable F1 midpoint (Hz)	787.3 (12.1)	755.8 (7.3)
1^st^ syllable F2 midpoint (Hz)	1961.2 (13.6)	1778.0 (9.0)
Total target word duration (ms)	844.1 (2.8)	846.3 (2.8)

Standard error of the mean in brackets. Significant (*p* < 0.05) differences between low- and high-competition words indicated in bold.

#### 2.1.2 Lexical statistics

The number of word onset competitors was calculated based on the first two phonemes of words (consonant-vowel) in the English Lexicon Project [1]. Unpaired-samples *t*-tests showed that low and high competition words were significantly different with regard to lexical competition (*t* = 12.865, *df* = 65.327, *p* < 0.001) and number of phonological neighbours (*t* = 2.153, *df* = 106.27, *p* = 0.03) [1]. High-competition words had on average almost five times as many possible word continuations (*M* = 290.9, *SD* = 133.7) as low-competition words (*M* = 61.2, *SD* = 31.0).

For the low and high competition word pairs, we controlled for word frequency in SUBTLEX-US [33] and SUBTLEX-UK [34], as well as word-average biphoneme, triphoneme and positional phonotactic probability through IPHOD [35], imageability [36], age of acquisition [37,38] and concreteness [39]. An analysis of variance showed that there were no differences in word class between low and high competition words (*F*(3,10) = 0.242, *p* = 0.865), with 26 words primarily used as nouns in the low-competition group and 24 in the high-competition group (e.g., *money*). There were 19 words primarily used as verbs in the low-competition group and 22 in the high-competition group (e.g., *cherish*), 14 adjectives in the low-competition group and 13 in the high-competition group (e.g., *jealous*), as well as one adverb in each of the groups (*never* and *circa*). We further controlled for acoustic characteristics, including F0, F1 and F2 frequency of the initial syllable, as well as intensity and target word duration (Table 1).

### 2.2 Experimental procedure

Twenty right-handed native monolingual speakers of Australian English (mean age = 21.4 years, *SD* = 3.6 years, range 18–30 years, 14 female) participated in the study after providing written consent. The study was approved by the Western Sydney University Human Research Ethics Committee (H11022). None reported neurological impairment or impaired hearing. The experiment was conducted using E-Prime 2 software [40] in a dimly lit, electrically shielded room. In a two-alternative forced-choice lexical decision task, participants pushed the right or left button on a button box to indicate whether the word was a real word in English or not. There were four blocks in total, and participants were encouraged to take a short break between blocks. The button order was counter-balanced across blocks and participants. The inter-trial interval varied randomly between 2000 and 3000 milliseconds. Stimuli were presented binaurally using Etymotic ear-tube insert earphones at a comfortable volume kept constant for all participants. Mean experimental duration was 28 minutes.

### 2.3 EEG recording and data pre-processing

A BioSemi ActiveTwo 64-channel system was used to record EEG data referenced to CMS online at a sample rate of 5 kHz. Electrode offset was kept below ±50 mV. The EEG data was pre-processed using EEGLAB (version 2020.1) [41] in MATLAB (version 9.9 R2020b). Data was re-referenced to average mastoids and downsampled to 250 Hz offline. A finite impulse response (FIR) high-pass filter of 0.01 Hz (cut-off frequency 0.005 Hz (-6 dB)) and a FIR low-pass filter of 30 Hz (cut-off frequency 33.75 Hz (-6 dB)) were applied to the continuous data.

Electrooculogram (EOG) electrodes were placed at left and right outer canthi, as well as above and below the right eye. Ocular artefacts were identified and manually removed using independent components analysis (ICA) [42]. After ocular component rejection, epochs with amplitudes exceeding ±100μV were discarded (average 10% trial rejection rate). A 200-millisecond time window before onset of the critical stimulus was used for baseline correction.

EEG data was analysed at two separate time points: first-syllable onset (500 ms epoch) and second-syllable onset (800 ms epoch). These epochs were chosen based on previous literature, so that the first window aimed to capture differences in first-syllable ERP amplitudes modulated by lexical competition (pre-activation negativity [5–7,9,13,14]), while the second window was used to capture a pseudoword N400 effect, with an expected peak between 300–500 ms [43]. The N400 analysis was chiefly included to indicate that pseudowords were perceived as such, i.e., eliciting larger N400 amplitudes than real words [43].

Nonparametric cluster-based permutation analyses were carried out using FieldTrip (version 20181119) [44]. Significance probability was calculated using the Monte Carlo method (cluster-forming alpha = 0.05, permutation alpha = 0.025, minimum number of electrodes required for a cluster = 2, randomisations = 5000).

## 3. Results

### 3.1 Behavioural results

Response times (RT) were measured from target word onset and analysed using a generalised linear mixed-effects model with an inverse Gaussian function and identity link [45] using the lme4 package in R [46]. Competition (low/high) and Lexicality (real/pseudo) were included as deviation-coded fixed effects (Competition low = 1, high = -1, Lexicality real = -1, pseudo = 1), with participant and item as random effects. To reduce any effects of outliers, trials above and below 2 standard deviations from the mean were removed before the analysis (7.1% of trials).

For the RT analysis, the maximal model with random intercept and slope for participant and item revealed an effect of Lexicality (*p* < 0.001, see Table 2 for details). RTs were faster for real words (*M* = 1436 ms (*SD* = 429 ms)) than for pseudowords (*M* = 1535 ms (*SD* = 440 ms)) (Table 3). As a follow-up, a model without fixed effects was compared to the maximal generalised mixed-effects model, showing the latter to be a better fit to the data (ΔAIC = -1077.7, *p* < 0.001).

**Table 2:** Fixed-effects estimates: Response time.

	*β*	*SE*	*t*	*p*
Intercept	1805.35	24.63	73.296	< 2e-16
Competition group	-20.72	13.48	-1.537	0.124
Lexicality	-78.10	16.69	-4.679	2.89e-06
Competition group * Lexicality	10.82	11.16	0.970	0.332

Fixed-effects estimates from the linear mixed-effects model of response times.

**Table 3:** Response time: Mean and standard deviation values per condition.

	RealLo	RealHi	PseudoLo	PseudoHi
Mean	1429 ms	1444 ms	1505 ms	1565 ms
Standard deviation	433 ms	426 ms	429 ms	448 ms

Mean and standard deviation values for response time per condition.

Response accuracy was analysed using a logistic mixed-effects model with a maximal random-effects structure identical to that of the RT analysis. After this model failed to converge, the random-effects structure was iteratively simplified. A model with random intercepts for participant and item revealed an effect of Lexicality (*p* = 0.01, see Table 4 for details), with better response accuracy for pseudowords (*M* = 90,8% (*SD* = 38,6%)) as compared to real words (*M* = 81,8% (*SD* = 28,9%)) (Table 5).

**Table 4:** Fixed-effects estimates: Response accuracy.

	*β*	*SE*	*t*	*p*
Intercept	3.2269	0.2234	14.442	<2e-16
Competition group	0.1384	0.1769	0.783	0.4338
Lexicality	-0.4522	0.1770	-2.556	0.0106
Competition group * Lexicality	-0.3253	0.1774	-1.834	0.0666

Fixed-effects estimates from the linear mixed-effects model of response accuracy.

**Table 5:** Accuracy: Mean and standard deviation values per condition.

	RealLo	RealHi	PseudoLo	PseudoHi
Mean	80.3%	83.2%	93.3%	88.2%
Standard deviation	39.8%	37.4%	25.0%	32.3%

Mean and standard deviation values for response accuracy per condition.

### 3.2 EEG results

**Fig 1:**
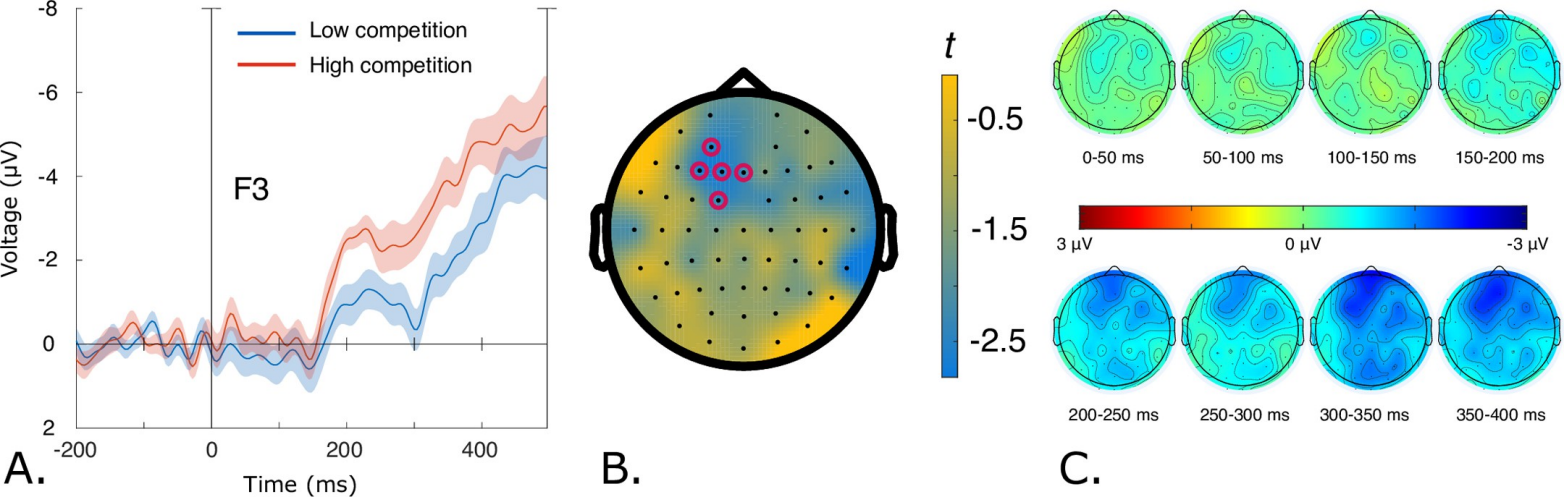
A. Left frontal first-syllable negativity in response to high-competition real words at electrode F3 (middle of cluster). The zero-point is at word onset. Shaded areas indicate standard error of the mean. Negative values are plotted up. B. Left-lateralised cluster extent indicated by red circles. C. Effect topography maps (high competition words minus low competition words) between 0–400 ms in 50-millisecond increments.

Planned comparisons found no effect of Competition for pseudowords at first-syllable onset. However, for real words the cluster-based permutation test revealed a difference between words with low and high competition (*p* < 0.05, Cohen’s *d* = 0.58). This corresponded to a negative cluster in the data for high competition words beginning at around 150 milliseconds after word onset. This cluster was most pronounced over left-anterior electrodes (see Fig 1). An analysis of subject variability in mean amplitudes over the identified cluster of electrodes (AF3, F1, F3, FC1 and Fz) in a 150–400 ms time window from word onset showed that 70% of participants displayed more negative amplitudes to real word onsets with more lexical competitors.

**Fig 2:**
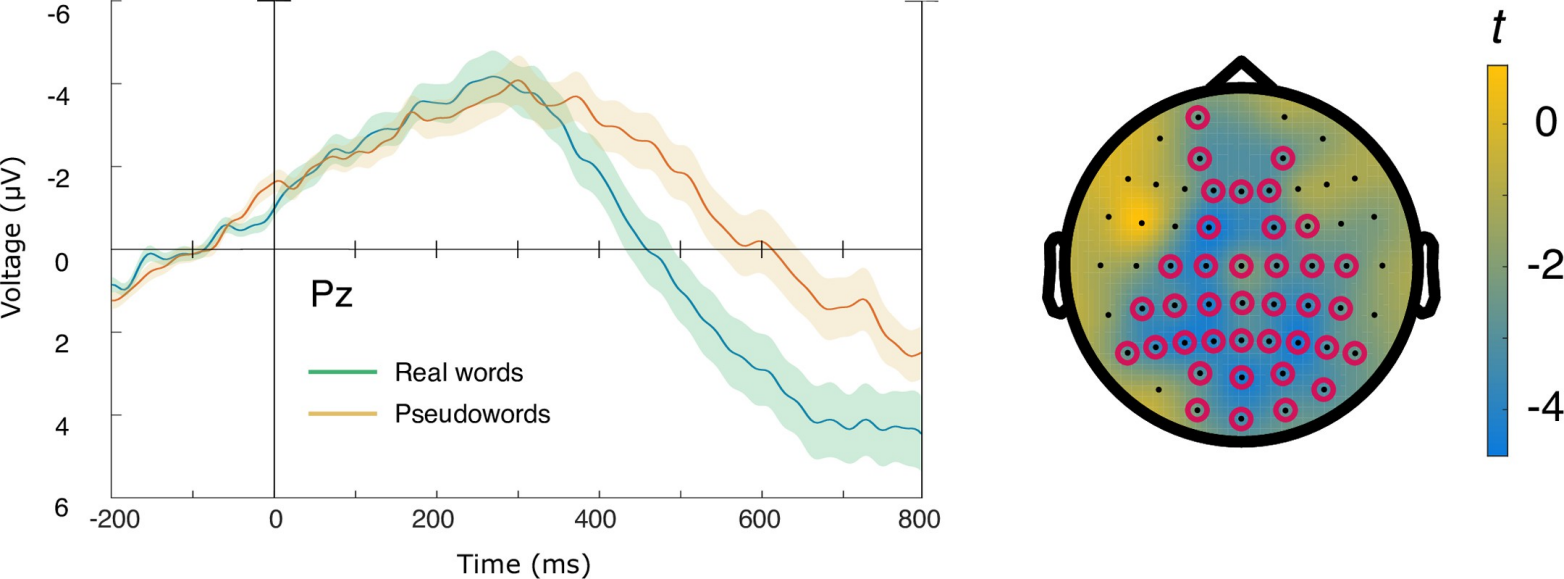
Left: Second-syllable N400 effect for pseudowords at electrode Pz. Shaded areas indicate standard error of the mean. Negative values are plotted up. Right: Cluster extent indicated by red circles.

At second-syllable onset, a main effect of Lexicality (N400) was found (*p* < 0.01, Cohen’s *d* = 0.72), with a cluster beginning at around 300 milliseconds. No main effect of Competition was found. The Lexicality cluster was broadly distributed across centro-posterior electrodes (see Fig 2). This indicates that pseudowords were generally processed as such [43].

## 4. Discussion

We investigated the effect of lexical competition on the neural processing and recognition of spoken English words. Real word onsets with more lexical competitors elicited increased pre-activation negativities (PrAN)–as compared to word onsets with fewer competitors–over left-anterior channels beginning around 150 ms from word onset, corroborating our hypothesis that a pre-activation negativity modulated by lexical competition would be found for English words.

The main difference from pre-activation negativities previously found in Swedish and Danish studies [5–10,12–14] is that the direction of the effect in the present study is reversed: more lexical competitors at word onset led to an increased negativity. In this respect, the results can be compared to one previous study of neighbourhood density in English [27], which, however, showed different topographical distributions of the ERP effects. We argue that the PrAN found in the present study reflects a process where the probability of a successful lexical match changes as a function of lexical competition as a word unfolds [5–10,13]. The reversal of the direction of the effect in comparison with previous Swedish studies may be explained by differences in language structure and experimental task paradigms. As for language structure, suprasegmental features in Swedish–tones–can play a role in lexical competition analogously to segments in languages such as English. Thus, two segmentally identical Swedish word onsets can differ greatly in how many potential continuations they cue, based on whether the onset is associated with a low or high tone. For example, consider the Swedish words *buren*_*H*_ (‘carried’) and *buren*_*L*_ (‘the cage’). The words are segmentally identical, have identical phonotactic probability, and differ only in word stem tone. Importantly, the tonal difference alone means that the word onset *bu-* with a high tone leads to a tenfold increase in lexical competition in the first two phonemes [14]. Word onsets with fewer possible continuations have been found to elicit larger left-frontal ERP negativities in Swedish. In English, lexical competitors differ only at the segmental level: *co-* in *cobble* leads to more possible word continuations than *go-* in *gobble* (*co-* has almost 9 times as many possible continuations in the English Lexicon Project corpus [1]). Word onsets with more competitors (such as *co-* in *cobble*) gave rise to larger left-frontal pre-activation negativities in the present study. It is possible that the experimental task plays an important role in the reversal of the effect direction of the pre-activation negativity between Swedish and English. In Swedish, the most commonly used task has been to judge whether a word is in singular or plural in the case of nouns, and present or past tense in the case of verbs. By implicitly being asked to guess which ending the word will have (i.e., a suffix marking number or tense), participants are encouraged to predict the word ending as quickly as possible, using the word stem tone as a clue. Word onsets with ten times fewer lexical competitors will therefore increase a listener’s confidence as regards the identity of the upcoming suffix, something which has been found to occur even in cases where the word stem itself carries no semantic meaning [10,13]. This is different from a lexical decision task, where participants judge whether a stimulus is a real word. As suggested previously, increased lexical competition leads to a larger number of co-activated words [27], increasing the listener’s certainty that the unfolding stimulus is in fact an existing lexical item, meaning that competition in the first two phonemes may have a facilitatory effect in a lexical decision task, at least at the early, neuroelectric level. That study found the same effect–albeit weaker–in a semantic decision task, meaning that it was relatively task invariant. This pattern of task invariability was also found by Hunter [22], where neighbourhood effects were found in both a lexical decision task and a same-different task, but with a larger statistical effect size in the former.

No significant ERP effect was found for pseudoword onsets in the present study. This may have been due to the constraints placed on the creation of the rhyming pseudoword pairs, such that some may have remained potential lexical candidates for longer than others, potentially cancelling out the effect.

Interestingly, behavioural response accuracy in the present study was lower for real words, indicating that participants may have found the task difficult. This is also reflected in the overall long response times (*M* = 1485 ms, *SD* = 437 ms). While it may not have been possible to detect fine-grained differences in behavioural responses to low- and high-competition words, sub-lexical facilitation may still be reflected early on at the neuroelectric level within 200 ms of the onset of the word. For example, task difficulty could manifest itself such that a listener may not have the word *zealous* (the onset of which has 11 times fewer possible word continuations than *jealous*) in their lexicon, or it is not readily available to them. Lexical competition at word onset may thus not influence the behavioural response to that type of item in a lexical decision task, but it does not rule out an effect at the sub-lexical or neurophysiological level.

Future research and experimental paradigms will have to elucidate the effects of task and context on this early ERP negativity, but also its effect on subsequent brain potentials in response to violated expectations at the phonemic and lexical levels, something which was not included in the present study. If an increased negativity reflects increasing strength in the updating of one’s beliefs, we might expect associated subsequent increases in e.g. MMN, P300 or N400 amplitudes in response to violations at different (but word-internal) levels [47]. Thus, it might be possible in future research to indirectly interrogate the drivers of PrAN in relation to different tasks by investigating the brain’s belief updating through subsequent mismatch responses at different levels of the inference hierarchy [47]: from the MMN at the level of acoustic features or the phoneme [48], the P300 for context violations [49,50] to the N400 for semantic violations [43]. Different mismatch components have previously been found in response to invalid tone-suffix combinations in Swedish and Danish studies. For example, in paradigms where the ending of the word is replaced by a cough, the neurophysiological mismatch response (P3a) to the cough has been found to correlate with PrAN amplitude on the preceding word stem: listeners are more surprised by a replaced suffix when they have committed more strongly to the word ending [13]. Increased PrAN amplitudes on the word onset have also been found to correlate with subject variability in response accuracy, with more accurate participants displaying larger ERP negativities [13]. Mismatching tone-suffix combinations have also led to different subsequent mismatch ERP components: left-anterior negativity (LAN) [13,29], N400 [6,10,12,28] and P600 [5,6,8–10,12,13,28,29]. This suggests that mismatching word endings are surprising–potentially at different levels of linguistic representation (morphological in the case of LAN [51,52] and semantic in the case of N400 [43])–leading to reanalysis, as reflected in the P600 [53].

To tie together the previous and present results, we note that, while the paradigms differ in experimental task design and language structure, there are also commonalities between them. In both lexical decision tasks and the Swedish tasks–which are more explicitly predictive–word onsets can be more or less useful for completing the task successfully. In both types of paradigms, as the word unfolds, listeners’ belief in the success or outcome of a specific response (word/non-word in a lexical decision task, and singular/plural suffix in a morphological task) increases or decreases depending on factors such as lexical competition, something which is reflected in the amplitude of the pre-activation negativity. In a lexical decision task, given a word onset in a dense neighbourhood, the listener is afforded more certainty that the unfolding item is a real word. Similarly, in the Swedish paradigm, reduced competition in the first syllable allows the listener to commit more strongly to the word ending. In a more prediction-oriented English-language paradigm, it is indeed possible that early ERP negativity amplitudes would increase for word onsets with fewer competitors, if this factor would help listeners predict the word ending: an operation which may be relatively unnecessary when performing a speeded lexical decision task, but useful–for example–when listening to speech in adverse conditions. More research is thus needed to investigate the impact of task demand and design on early ERP effects of spoken-word recognition.

Across the English, French, Swedish and Danish paradigms, this type of predictive decision-making is perhaps best described by the rapid peaking or narrowing of the probability distribution–or entropy reduction–of possible word endings or response outcomes in accordance with principles of Bayesian inference [3,54]. Thus, it is not an argument that listeners necessarily entertain hypotheses about specific outcomes at this level of processing (phonemic or lexical), but rather that factors such as lexical competition can shrink the decision space enough to rapidly increase a listeners’ certainty that a response will be successful, resulting in an increased left-anterior ERP negativity beginning around 150 ms after word onset: the pre-activation negativity [14]. In other words, priors are becoming peaked around the most likely word or words [54]. First-syllable lexical competition may also carry different predictive information depending on the nature of the task or context in which a word is heard–just like different phonological cues carry different weight in different languages–and this information can be used to update the beliefs and reweight the hypotheses that are used to infer what the unfolding word is, given the signal. In the present study, word onsets with more competitors facilitated spoken-word recognition. We propose that the pre-activation negativity can be used as a tool to understand the early stages of lexical prediction and recognition in the brain, helping us uncover the drivers and cues that enable efficient speech processing across languages.

## Supporting information

S1 File List of stimulus words. (DOCX)
