# Supplementary material for: Early neuro-electric indication of lexical match in English spoken-word recognition
Source: PLoS One. 2023 May 18;18(5):e0285286. doi: 10.1371/journal.pone.0285286 (PMC10194953; doi:10.1371/journal.pone.0285286)
Supplement: S1 File — (DOCX) [file pone.0285286.s001.docx]

Appendix. Stimulus words.

| Word no | Word |
| --- | --- |
| 1 | gobble |
| 2 | cobble |
| 3 | nickel |
| 4 | sickle |
| 5 | locket |
| 6 | pocket |
| 7 | thicket |
| 8 | picket |
| 9 | jingle |
| 10 | single |
| 11 | toddle |
| 12 | coddle |
| 13 | volley |
| 14 | collie |
| 15 | felon |
| 16 | melon |
| 17 | venue |
| 18 | menu |
| 19 | poodle |
| 20 | noodle |
| 21 | shallow |
| 22 | callow |
| 23 | jumble |
| 24 | bumble |
| 25 | joggle |
| 26 | boggle |
| 27 | nether |
| 28 | tether |
| 29 | waggle |
| 30 | haggle |
| 31 | kitten |
| 32 | mitten |
| 33 | wacky |
| 34 | lackey |
| 35 | lotto |
| 36 | motto |
| 37 | hinder |
| 38 | cinder |
| 39 | kettle |
| 40 | settle |
| 41 | thistle |
| 42 | missile |
| 43 | tussle |
| 44 | bustle |
| 45 | gibbon |
| 46 | ribbon |
| 47 | shamble |
| 48 | ramble |
| 49 | curtain |
| 50 | certain |
| 51 | vassal |
| 52 | hassle |
| 53 | nibble |
| 54 | dibble |
| 55 | daddy |
| 56 | caddie |
| 57 | wangle |
| 58 | mangle |
| 59 | yeti |
| 60 | petty |
| 61 | tunny |
| 62 | money |
| 63 | chatter |
| 64 | matter |
| 65 | chuckle |
| 66 | suckle |
| 67 | yellow |
| 68 | mellow |
| 69 | shoddy |
| 70 | body |
| 71 | never |
| 72 | sever |
| 73 | shovel |
| 74 | hovel |
| 75 | zealous |
| 76 | jealous |
| 77 | vandal |
| 78 | candle |
| 79 | vapid |
| 80 | rapid |
| 81 | valid |
| 82 | pallid |
| 83 | Yemen |
| 84 | lemon |
| 85 | valley |
| 86 | rally |
| 87 | nectar |
| 88 | rector |
| 89 | beagle |
| 90 | regal |
| 91 | cherish |
| 92 | perish |
| 93 | nestor |
| 94 | pester |
| 95 | number |
| 96 | cumber |
| 97 | nappy |
| 98 | happy |
| 99 | sherry |
| 100 | berry |
| 101 | German |
| 102 | sermon |
| 103 | narrow |
| 104 | marrow |
| 105 | nestle |
| 106 | pestle |
| 107 | shackle |
| 108 | cackle |
| 109 | zealot |
| 110 | pellet |
| 111 | Gurkha |
| 112 | circa |
| 113 | nervous |
| 114 | service |
| 115 | yonder |
| 116 | ponder |
| 117 | Yankee |
| 118 | lanky |
| 119 | whopper |
| 120 | copper |
| 121 | gottle |
| 122 | cottle |
| 123 | nithel |
| 124 | sithle |
| 125 | loddet |
| 126 | poddet |
| 127 | thibbet |
| 128 | pibbet |
| 129 | jintle |
| 130 | sintle |
| 131 | tommle |
| 132 | commle |
| 133 | vobbey |
| 134 | cobbie |
| 135 | femon |
| 136 | memon |
| 137 | velue |
| 138 | melu |
| 139 | poogle |
| 140 | noogle |
| 141 | shannow |
| 142 | cannow |
| 143 | juntle |
| 144 | buntle |
| 145 | jonnle |
| 146 | bonnle |
| 147 | nesser |
| 148 | tesser |
| 149 | waddle |
| 150 | haddle |
| 151 | kiffen |
| 152 | miffen |
| 153 | watty |
| 154 | lattey |
| 155 | lonno |
| 156 | monno |
| 157 | himber |
| 158 | cimber |
| 159 | kevvle |
| 160 | sevvle |
| 161 | thirrle |
| 162 | mirrle |
| 163 | tuggle |
| 164 | buggle |
| 165 | gickon |
| 166 | rickon |
| 167 | shantle |
| 168 | rantle |
| 169 | curkain |
| 170 | cerkain |
| 171 | vammal |
| 172 | hammle |
| 173 | nirrle |
| 174 | dirrle |
| 175 | davvy |
| 176 | cavvie |
| 177 | wample |
| 178 | mample |
| 179 | yeki |
| 180 | pecky |
| 181 | tussy |
| 182 | mossey |
| 183 | chabber |
| 184 | mabber |
| 185 | chubble |
| 186 | subble |
| 187 | yeppow |
| 188 | meppow |
| 189 | sholly |
| 190 | bolly |
| 191 | nepper |
| 192 | sepper |
| 193 | shonel |
| 194 | honel |
| 195 | zeakous |
| 196 | jeakous |
| 197 | vantal |
| 198 | cantle |
| 199 | vathid |
| 200 | rathid |
| 201 | vanid |
| 202 | pannid |
| 203 | Yeggen |
| 204 | legon |
| 205 | vappey |
| 206 | rappy |
| 207 | neclar |
| 208 | reclor |
| 209 | beaple |
| 210 | repal |
| 211 | chedish |
| 212 | pedish |
| 213 | neskor |
| 214 | pesker |
| 215 | nunger |
| 216 | cunger |
| 217 | nassy |
| 218 | hassy |
| 219 | sheppy |
| 220 | beppy |
| 221 | Gerdan |
| 222 | serdon |
| 223 | nackow |
| 224 | mackow |
| 225 | neffle |
| 226 | peffle |
| 227 | shathle |
| 228 | cathle |
| 229 | zeatot |
| 230 | pettet |
| 231 | Gurpha |
| 232 | cirpa |
| 233 | nerlous |
| 234 | serlice |
| 235 | yonler |
| 236 | ponler |
| 237 | Yantee |
| 238 | lanty |
| 239 | whodder |
| 240 | codder |
